# Enhancing construction safety: predicting worker sleep deprivation using machine learning algorithms

**DOI:** 10.1038/s41598-024-65568-2

**Published:** 2024-07-08

**Authors:** S. Sathvik, Abdullah Alsharef, Atul Kumar Singh, Mohd Asif Shah, G. ShivaKumar

**Affiliations:** 1 Department of Civil Engineering, Dayananda Sagar College of Engineering, Bengaluru, Karnataka 560111 India; 2 Department of Civil Engineering, College of Engineering, King Saud University, P.O. Box 800, 11421 Riyadh, Saudi Arabia; 3 Department of Civil Engineering, College of Engineering and Technology, SRM Institute of Science and Technology, Kattankulathur, Tamil Nadu 603203 India; 4 Kabridahar University, P.O Box 250, Kebri Dehar, Ethiopia; 5 Division of Research and Development, Lovely Professional University, Phagwara, Punjab 144001 India; 6 Centre of Research Impact and Outcome, Chitkara University Institute of Engineering and Technology, Chitkara University, Rajpura, Punjab 140401 India

**Keywords:** Sleep deprivation, Machine learning, Construction safety, Construction workers, Safety performance, Civil engineering, Engineering

## Abstract

Sleep deprivation is a critical issue that affects workers in numerous industries, including construction. It adversely affects workers and can lead to significant concerns regarding their health, safety, and overall job performance. Several studies have investigated the effects of sleep deprivation on safety and productivity. Although the impact of sleep deprivation on safety and productivity through cognitive impairment has been investigated, research on the association of sleep deprivation and contributing factors that lead to workplace hazards and injuries remains limited. To fill this gap in the literature, this study utilized machine learning algorithms to predict hazardous situations. Furthermore, this study demonstrates the applicability of machine learning algorithms, including support vector machine and random forest, by predicting sleep deprivation in construction workers based on responses from 240 construction workers, identifying seven primary indices as predictive factors. The findings indicate that the support vector machine algorithm produced superior sleep deprivation prediction outcomes during the validation process. The study findings offer significant benefits to stakeholders in the construction industry, particularly project and safety managers. By enabling the implementation of targeted interventions, these insights can help reduce accidents and improve workplace safety through the timely and accurate prediction of sleep deprivation.

## Introduction

Sleep deprivation (SD), characterized by frequent interruptions in breathing during sleep owing to increased blockage of the airways, affects 7% of female and 20% of male employees^1^. Apart from excessive daytime tiredness and frequent awakenings, the most common symptom of obstructive sleep apnea (OSA) is snoring. Moreover, hypertension, atherosclerosis, coronary heart disease, and vasculitis are established to be linked to this ailment^2,3^. Furthermore, SD is used to describe polygenic illness mellitus, hypoglycemic agent resistance, dyslipidemia, obesity, and psychological abnormalities^4^.

Early detection of this disease is essential given its prevalence in various parts of the world, including South Indian states, and its substantial impact on individuals’ health and quality of life^5,6^. According to the international diagnostic criteria, patients with symptoms of SD and an apnea–hypopnea index (AHI) between 5 and 15/h are diagnosed with SD as a disorder of the central nervous system^7^. The diagnosis of sleep apnea necessitates a comprehensive polysomnography (PSG) test. Nonetheless, due to its high cost, clinical practitioners often reserve this test to select patients^8,9^.

Numerous studies have explored the development of sleep deprivation (SD) and apnea–hypopnea index (AHI) prediction models tailored to individuals likely affected by these conditions. These models incorporate a grading system that does not require polysomnography^9,10^. Regression analyses were conducted, accounting for demographic variables such as age and sex and incorporating measurements of factors such as body mass index and waist or neck size^11,12^. However, prior prediction model studies have demonstrated that construction workers’ country, ethnicity, and characteristics significantly influence the results^13,14^. For instance, SD is common in East Asians, including Indians, Chinese, and Japanese, and even in non-obese individuals^14,15^. One particular reason is their narrower cranial traits^14,15^. Consequently, several ethnic groups should be tested to ascertain the applicability of similar prediction models to populations including Indians. Recent advancements in computational capabilities have significantly increased the reliance of predictive modeling approaches on machine learning techniques.

Machine learning enables computers to learn from real-world data and discover previously unknown patterns^16–18^. Traditional data analysis methods often rely on subjective opinions with analysts choosing specific methodologies. In contrast, machine learning progressively improves results over time through iterative processes^19,20^. For example, precisely defining diseases for identification using mathematical models poses a significant challenge in the medical field. Machine learning is particularly applicable in data-rich sectors of medicine, which require extensive data for learning, processing, training, and validation^21,22^. Consequently, various machine learning algorithms can be utilized to achieve specific objectives. Common classical machine learning models include logistic regression (LR), support vector machine (SVM), random forest (RF), and decision tree (DT). These methods are categorized as supervised learning that uses labeled data^23^.

Previous studies on prediction models for the Indian population have predominantly utilized multivariate analysis or support vector machine (SVM). One study on sleep deprivation (SD) in construction workers utilized logistic regression (LR) with data from 433 individuals, achieving a sensitivity of 74.6% and a specificity of 66.3%^24–26^. Another study employed an SVM to create an SD prediction model based on data from 566 individuals, resulting in an accuracy rate of 84.15%^27^. Apart from regression analysis and SVM, other prediction methods have been utilized by several researchers due to the increase in computational power along with the ability to conduct the analysis in the cloud environment. The rationale for employing machine learning techniques in this study is multifaceted. First, machine learning algorithms excel at identifying intricate patterns and relationships within complex datasets, making them well-suited for the analysis of multidimensional data associated with sleep deprivation. Secondly, these techniques can handle a wide range of input variables, enabling the incorporation of diverse factors that may influence sleep patterns, such as physiological, environmental, and behavioral variables. Furthermore, machine learning models have the ability to learn and adapt dynamically, allowing for continuous refinement and improvement as new data becomes available. This adaptive nature is particularly advantageous in the context of sleep deprivation, where individual variations and evolving circumstances necessitate flexible and responsive models. Additionally, it is worth noting that no machine learning-based prediction models have been developed to predict SD among South Indian construction workers. The primary objective of this study was to evaluate the performance of various machine learning algorithms in predicting sleep deprivation among construction workers. In addition to validating the feasibility of predicting sleep deprivation, this study assessed the predictive efficacy of different machine learning algorithms. The remainder of this paper is structured as follows: section two outlines the research methodology. Section three presents the results, followed by section four which offers a discussion of these findings. Finally, section five concludes the paper with a comprehensive summary of the study's key outcomes.

## Methodology

**Figure 1:**
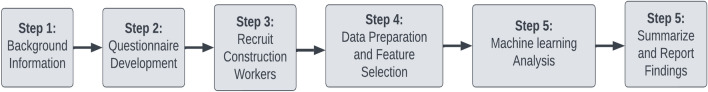
Research Methodology of the study.

To accomplish the study's aims and objectives, the authors employed a multifaceted research methodology. Figure 1 provides a summary of the adopted research methodology. The subsequent sub-sections offer detailed explanations of the implemented research approach.

### Data collection

In this study, a total of 295 construction workers were selected from the SRM Medical College and Research Centre, located in Tamil Nadu, India, for the collection of relevant data. The cohort consisted exclusively of Indian construction workers, each exhibiting symptoms of sleep deprivation (SD) such as daytime sleepiness, frequent snoring, and cases of witnessed sleep apnea. These symptoms were critical in identifying the target group for this study. A detailed analysis was conducted to further understand the extent of the SD among these workers. This analysis involved correlating 92 different variables with workers' SD and overall sleep status, providing a comprehensive view of the factors influencing their sleep health. The data collection process adhered to rigorous protocols to ensure the reliability and validity of the gathered information. A comprehensive set of questionnaires was administered, the questionnaires were specifically tailored to the Indian context to ensure cultural relevance and accuracy. These included localized versions of well-established tools such as the Pittsburgh Sleep Quality Index (PSQI), Fatigue Severity Scale (FSS), and Epworth Sleepiness Scale (ESS). Furthermore, to complement the self-reported data, a series of anthropometric measurements was performed on the participants. These measurements were vital in providing baseline physical data, which could be crucial for understanding the correlation between physical characteristics and sleep disorders among workers. The entire data collection process was conducted in accordance with strict ethical guidelines, ensuring the protection of participants' rights and well-being.

The responses of 288 construction workers were rigorously analyzed, taking into account the exclusion of data from seven workers due to their incompleteness. The participating construction workers were systematically categorized into two distinct groups based on the presence or absence of sleep deprivation (SD): 220 construction workers were identified as experiencing SD, whereas the remaining 68 workers were classified as not suffering from SD. The methodology for this classification involved carefully defining the inclusion and exclusion criteria. Moreover, detailed anthropometric measurements were obtained, and polysomnography (PSG) tests were implemented on these workers to ensure accurate categorization and data collection. The study was conducted according to strict ethical guidelines. The Ethics Committee of SRM Medical College Hospital and Research Center (reference number 2186/IEC/2020) was thoroughly reviewed and approved for this research. This approval was contingent upon obtaining written consent from all participating construction workers, ensuring that they were fully informed about the nature of the study and their role in it. Such a process underscores the commitment to ethical standards and the importance of informed consent in conducting research that involves human subjects. This thorough approach not only reinforced the integrity of the research, but also ensured the protection and respect for the rights and well-being of the participants.

### Feature selection

**Figure 2:**
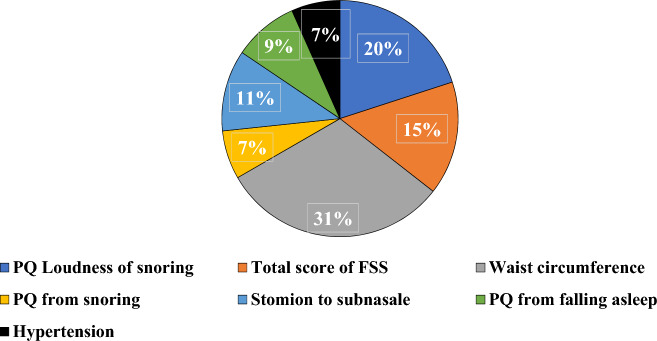
Significance of seven final features selected by the permutation algorithm.

The features of the model were selected using a permutation feature selection method involving 92 permutations^22^. This process began by training the model with all the variables. The importance of each feature was then evaluated based on the change in a specific performance score following a random shuffle of the feature's values^26^. Consequently, this shuffle disrupted the relationship between the feature and target outcome, revealing the significance of the feature^28^. Figure 2 shows the seven significance proportions of the final seven FSS features selected using the permutation algorithm. The seven variables are hypertension, stomion to subscale, snoring from the PQ, intensity of snoring from the PQ, total FSS score, waist circumference, and frequency of falling asleep from the PQ.

### Prediction of OSA using machine learning algorithms

Following a comprehensive review of the existing articles, the research team identified four widely employed machine learning algorithms to model the data effectively. These algorithms include logistic regression (LR), support vector machine (SVM), random forest (RF), and decision tree (DT). Each of these algorithms was chosen for its distinct methodological strength and suitability for the collected data. Prior to fitting the four identified models, the data were split into training and test sets. For each sleep disorder (SD) and non-sleep disorder (non-SD) group, 30% of the data were randomly chosen for testing (SD: 88 instances; non-SD: 25 instances), and the remainder for training (SD: 152 instances; non-SD: 49 instances). After completing the process, the four models (i.e., LR, DT, RF, and SVM) were trained.

LR is a widely used machine learning algorithm for classification tasks, such as determining if an email is spam or not^29^. It calculates probabilities to classify new observations into categories, excelling in situations in which data points relate linearly to these categories. It is commonly applied in healthcare, marketing, and finance, and is valued for its simplicity and ease of interpretation. Similarly, DT in machine learning is a commonly utilized classification approach that is primarily utilized for categorizing data. It functions similar to a flowchart, with each branch symbolizing a data-driven decision with a probability in a definitive categorization. Its popularity stems from its simplicity with which it can be understood and visually represented, making it a widely preferred choice for addressing a variety of classification issues across numerous industries and among researchers from different specializations. As for RF, it is a widely used for machine learning, which is known for its adaptability to complex data, while accounting for correlations and interactions among features. It is an ensemble learning tool that combines multiple decision trees to form a powerful classifier, thereby reducing the risk of overfitting.

The process began by transforming the input data into a high-dimensional space to establish an optimal boundary between the groups. Subsequently, an ensemble RF model was developed, which extended from multiple decision trees. The RF approach involves generating a series of decision trees and selecting the best classification based on their collective output. Additionally, the DT algorithm was employed to enhance the gradient boosting model (GBM). DT stands out for its faster execution and higher prediction accuracy compared to GBM. To prevent overfitting, a regularization function was incorporated into the model. Finally, a grid search was conducted to identify the most effective parameters for each machine learning model to ensure optimal performance^29^.

### Performance evaluation of machine learning models

The performance of LR and the other three machine learning techniques (SVM, RF, and DT) was assessed by calculating key metrics: specificity, sensitivity, positive predictive value (PPV), accuracy, and negative predictive value (PNV). These metrics were derived from the true-positive, false-positive (FP), true-negative, and false-negative (FN) outcomes of each model. In addition, the area under the curve (AUC) was computed to assess the overall performance of each model. The analysis was conducted in Python using the Scikit-learn library (version 0.23.2)^26^. The receiver operating characteristic (ROC) curves and their comparative analyses were performed using MedCalc software (version 14.0)^30^. The IBM SPSS for Windows was used for further statistical analyses^31^. To maintain the reliability of the findings, a statistical significance threshold was established at *p* < 0.05 to ensure the robustness and validity of the results.

### Ethics statement

This research was approved by the Ethics Committee of the SRM Hospital and Research Centre (2186/IEC/2020) and conducted according to the principles of the institutional ethical committee. The patients provided written informed consent to participate in the study. Informed consent was obtained from all matters belonging to this research study.

## Results

Table 1 presents a comparative profile of construction workers in the sleep disorder (SD) and non-sleep disorder (non-SD) groups. The reported statistics in Table 1 suggest that there is a statistically significant difference between the two groups in all cases, with a *p* value less than 0.05, except for sex and total sleep duration (min). The findings from the analysis suggest that sex is not a distinguishing factor between the OSA and Non OSA groups. Additionally, there was no difference in sleep duration between the two groups as well.

**Table 1:** Comparison of participant profiles in non-OSA and OSA groups.

Variables	OSA (N = 220)	Non OSA (N = 68)	*p* value
Age	46.3 ± 11.7	42.9 ± 12.3	0.0321*
Sex			0.071
Female	39 (14.6%)	14 (25.2%)	
Male	201 (87.4%)	60 (76.8%)	
Body mass index			0.0005*
0–18.4	4 (0.7%)	1 (0.1%)	
18.5–22.9	30 (13.1%)	25 (34.8%)	
23–24.9	51 (23.1%)	21 (27.4%)	
25–29.9	125 (51.7%)	23 (33.7%)	
≥ 30	30 (11.4%)	4 (4.6%)	
Hypertension	57 (28.5%)	7 (11.09%)	0.0106*
Total score ESS	11.1 ± 5.1	9.4 ± 5.5	0.016*
Total score FSS	36.3 ± 13.2	39.5 ± 13.4	0.071*
PSQI questionnaire			< 0.00001*
Intensity of snoring			
Intense than breathing	10 (3.3%)	14 (17.6%)	
Intense as talking	33 (12.9%)	21 (26.2%)	
Intense than talking	56 (26.5%)	17 (24.5%)	
Equal to neighboring rooms	141 (57.3%)	22 (31.7%)	
Frequency of snoring			0.0299*
Never	2 (0.7%)	4 (5.8%)	
0–1 time a week	3 (2.2%)	2 (2.4%)	
1–2 times a month	16 (6.3%)	7 (7.3%)	
2–3 times a month	22 (8.6%)	12 (14.3%)	
Almost every day	197 (82.2%)	49 (70.2%)	
Quit breathing			0.0103*
Never or nearly never	19 (9.2%)	9 (13.3%)	
0–1 time a week	12 (4.3%)	7 (8.2%)	
1–2 times a month	23 (12.4%)	11 (14.3%)	
2–3 times a month	58 (23.1%)	26 (36.1%)	
Nearly every day	128 (51.0%)	21 (28.1%)	
Measurements anthropometric			
Circumference neck (cm)			
< 29	5 (0.8%)	9 (8.6%)	< 0.0001*
29.1–34	41 (17.3%)	15 (19.1%)	
34.1–39	139 (52.6%)	42 (66.4%)	
39.1–44	47 (27.1%)	5 (5.2%)	
≥ 44.1	8 (2.2%)	3 (0.7%)	
Circumference waist (cm)			
< 69	6 (0.5%)	6 (4.2%)	< 0.0001*
69.1–79	21 (8.0%)	23 (28.4%)	
79.1–89	91 (40.4%)	45 (47.6%)	
89.1–99	86 (39.4%)	15 (12.7%)	
99.1–109	24 (8.9%)	7 (5.8%)	
109.1–119	12 (2.8%)	4 (2.3%)	
Stomion to subnasale (mm)			
< 19	3 (0.4%)	4 (3.5%)	0.0432*
19.1–24	62 (24.2%)	28 (39.8%)	
24.1–29	133 (59.4%)	34 (47.7%)	
29.1–35	35 (14.3%)	6 (7.6%)	
≥ 35	7 (1.7%)	2 (1.4%)	
Data polysomnography			
Total sleep duration (mins)	352.7 ± 56.7	351.4 ± 53.2	0.660
O_2_ desaturation index (number of events per hour)	29.3 ± 23.6	1.8 ± 1.3	< 0.001*

*Statistically significant.

**Figure 3:**
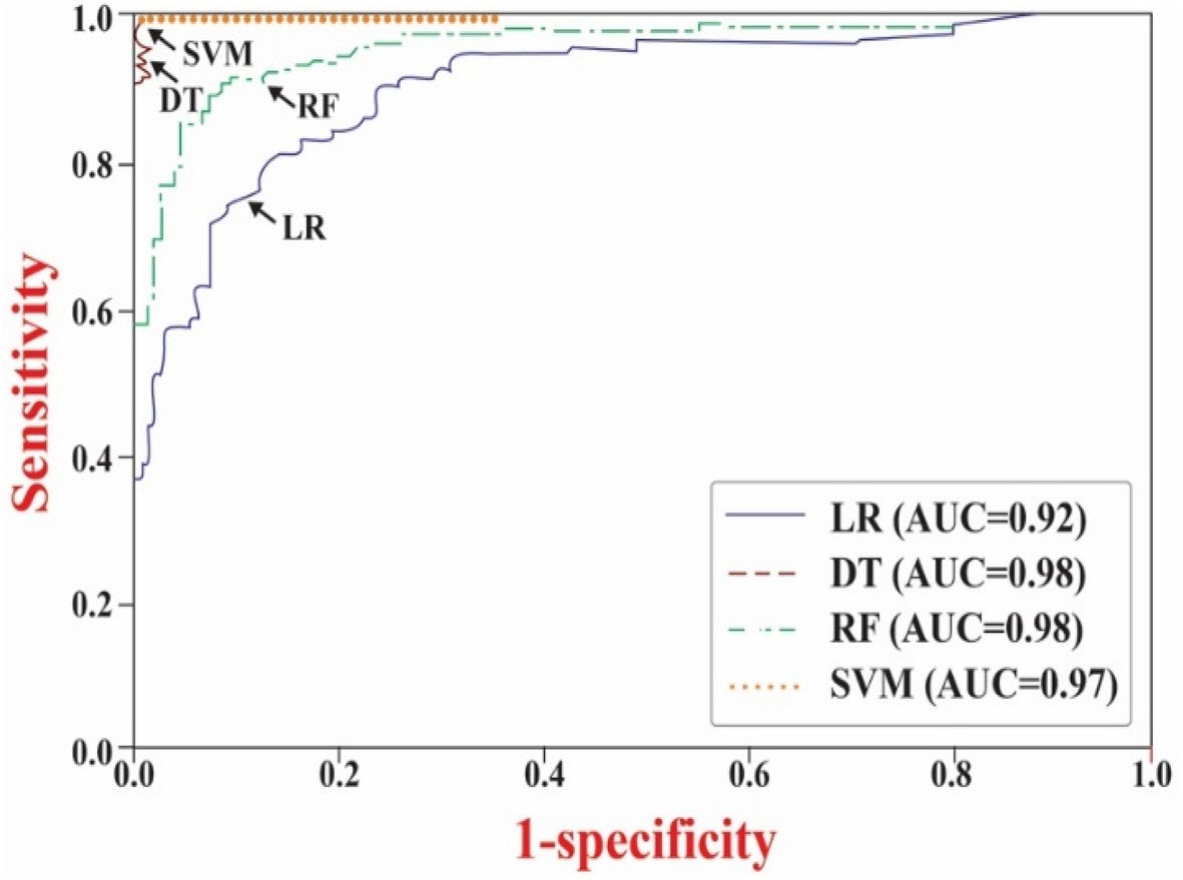
Comparison between various machine learning models of receiver operating characteristics for predicting OSA using the training dataset.

Each of the four machine learning models (i.e., LR, DT, RF, and SVM) was trained using seven variables to predict the SD. Additionally, their performance was evaluated using a designated set of test data, with the results are summarized in Table 2. The accuracy of the models in predicting SD was as follows: RF achieved 85.2% (confidence interval [CI] 78.7–89.5), SVM scored 99.4% (CI 97.8–97.8), DT attained 92.8% (CI 89.4–95.8), and LR recorded 99.3% (CI 96.7–99.8). The area under the curve (AUC) for these models, depicted in Fig. 3, was 0.95 (CI 0.89–0.97) for RF, 0.98 (CI 0.97–1.3) for SVM, 0.95 (CI 0.93–0.97) for DT, and 0.95 (CI 0.96–1.3) for LR. These results demonstrate the effective training of each model, indicating their proficiency in accurately predicting sleep disorders among construction workers.

**Table 2:** Comparison of participant profiles in non-OSA and OSA groups.

Models	(95% C.I) AUC	(95% C.I) Accuracy (%)	(95% C.I) Sensitivity (%)	(95% C.I) Specificity (%)	(95% C.I) PPV (%)	(95% C.I) PNV (%)
Dataset training						
LR	0.92 (0.88–0.95)	84.23 (79.16–88.28)	81.93 (74.67–87.38)	86.54 (79.19–91.72)	85.84 (80.11–90.31)	82.77 (77.25–87.23)
DT	0.98 (0.99–1.00)	99.04 (97.18–99.41)	97.72 (93.51–99.31)	98.35 (97.40–98.89)	98.33 (95.42–98.90)	97.78 (92.73–99.62)
SVM	0.97 (0.93–0.99)	91.78 (88.96–94.92)	91.78 (86.23–95.78)	91.80 (86.12–95.91)	91.78 (85.65–95.21)	91.78 (85.65–95.21)
RF	0.98 (0.97–0.99)	98.05 (96.45–99.27)	97.72 (93.51–99.88)	98.38 (94.51–98.43)	98.36 (93.32–98.04)	97.74 (92.59–98.61)
Dataset test						
LR	0.85 (0.79–0.92)	76.02 (65.47–84.91)	71.50 (58.5–82.7)	87.88 (66.6–98.4)	89.50 (77.74–96.88)	54.20 (35.47–71.29)
DT	0.88 (0.78–0.95)	84.45 (74.26–91.60)	81.41 (69.3–88.7)	87.88 (67.5–98.5)	89.61 (81.15–94.25)	70.60 (52.89–83.58)
SVM	0.83 (0.75–0.89)	79.61 (68.26–86.79)	71.50 (59.6–82.8)	87.88 (67.5–98.4)	87.51 (79.82–92.78)	61.02 (45.51–75.10)
RF	0.81 (0.76–0.90)	76.01 (68.41–84.88)	79.68 (67.4–89.4)	74.84 (52.3–88.5)	86.81 (78.61–92.34)	54.60 (40.65–68.50)

**Figure 4:**
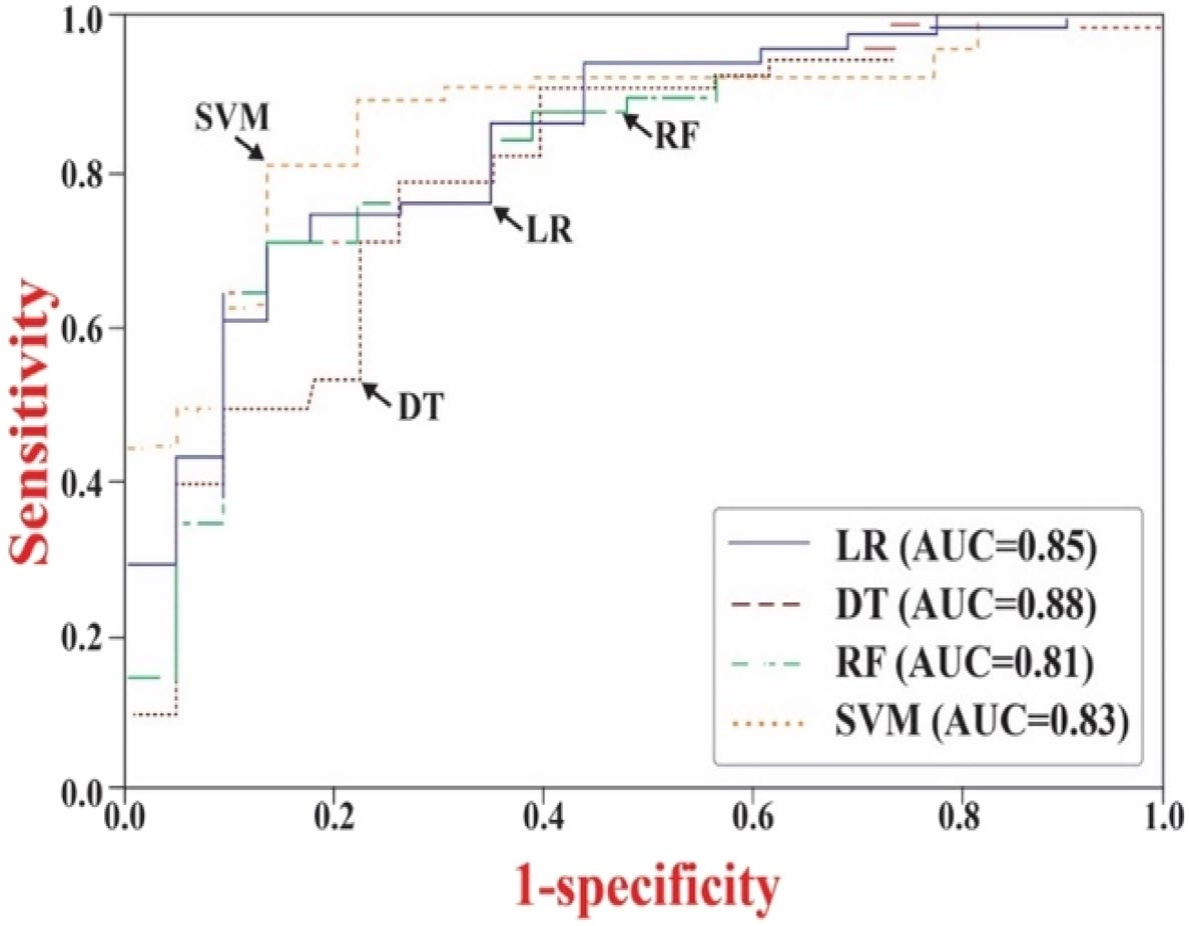
Comparison of characteristics of receiver operation between various machine learning models for the test dataset of OSA prediction.

The performance of sleep disorder (SD) prediction was assessed using a distinct set of test data. Among the models tested, the SVM demonstrated the highest accuracy. It achieved a sensitivity of 81.34% (CI 65.37–84.82), specificity of 87.97% (CI 68.6–99.4), positive predictive value (PPV) of 90.62% (CI 82.64–95.63), negative predictive value (PNV) of 69.77% (CI 52.99–83.85), and an overall accuracy of 85.45% (CI 75.64–92.70), as depicted in Fig. 4. In comparison, the DT model exhibited the lowest performance, with a sensitivity of 80.69% (CI 67.4–88.2), specificity of 74.92% (CI 52.7–90.2), PPV of 86.72% (CI 79.29–92.43), PNV of 54.58% (CI 38.57–68.05), and accuracy of 76.0% (CI 65.37–84.82). Notably, among the models, including SVM, RF, DT, and LR, SVM delivered the highest accuracy. In the Receiver Operating Characteristic (ROC) analysis, SVM also exhibited the highest area under the curve (AUC) at 0.88 (CI 0.78–0.94), followed by LR (0.85, CI 0.75–0.92), RF (0.83, CI 0.73–0.90), and DT (0.81, CI 0.71–0.89). Overall, the differences in the AUCs (*p* = 0.41) were statistically insignificant, indicating a comparable level of performance among the examined models.

**Figure 5:**
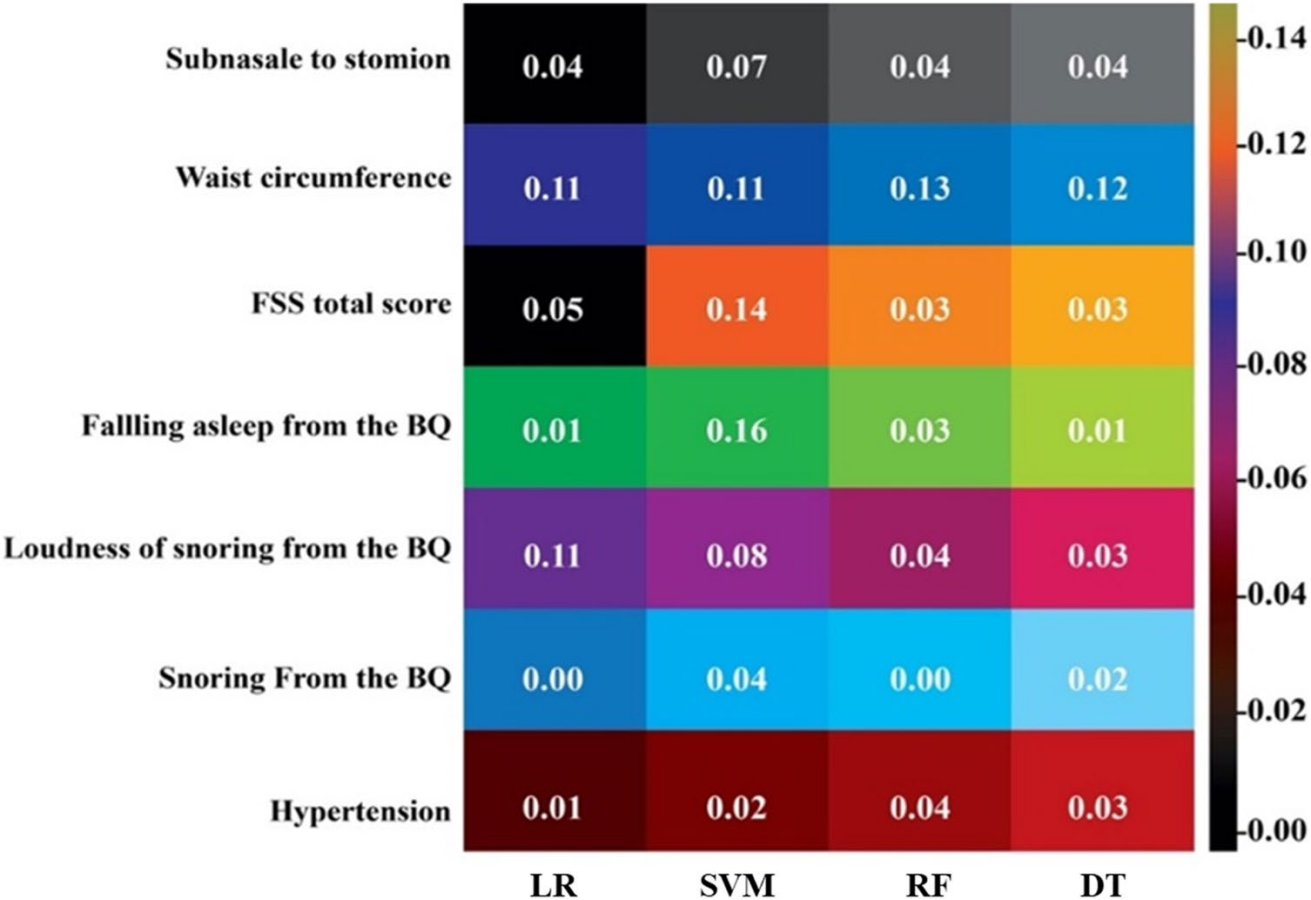
Heatmap of OSA prediction using machine learning algorithms for selected features.

Figure 5 presents a heatmap detailing the effect of each feature on sleep disorder prediction across the different machine learning models. Notably, waist circumference emerged as the most influential factor in SD prediction for all models, with importance scores of 0.15 in RF, 0.13 in SVM, 0.14 in DT, and 0.13 in LR. Additionally, the loudness of snoring, as measured by the PSQI, also significantly affected the SD prediction, with scores of 0.04 in RF, 0.09 in SVM, 0.05 in DT, and 0.13 in LR. Based on these findings, an application was developed for SD prediction that calculates the likelihood of SD by providing values for these seven key features. This application provides users with a probability estimate of having SD, leveraging the predictive power of the developed machine learning models as part of this study.

## Discussion

Based on the responses of 282 participants, this experiment selected 92 variables as key indices for predicting SD and compared the performance of several machine learning methods for SD prediction (LR, SVM, RF, and DT). As observed, seven indices affected the prediction of SD (hypertension, subscale of stomion, PSQI from snoring, snoring loudness from the PSQI, falling sleep from the PSQI, and total FSS score)^1,32^. Using the selected indices as inputs, these models predicted the SD with an accuracy of 88% (SVM (0.88), LR (0.85), SVM (0.83), RF (0.84), and DT (0.84)). This outcome was primarily due to foreign terrorist organizations (0.80). In terms of the supported accuracy (76.0%), SVM (84.44%) ranked first, followed by RF (79.68%), LR (76.0%), and DT (76.0%).

Overall, the PPV was considerably high and the PNV was low for most of the models tested in this experiment. According to the present findings, several models have exhibited few autonomous agencies and numerous FNs^24^. If the model predicts the occurrence of SD (SD group) even when a specific case does not exist, it is called FP, and if the model predicts the absence of SD (non-SD group) even when a specific case exists, it is called FN (SD group)^28^. If one of the two groups contains more training data than the other, the model training is likely to be biased toward the group with more data. As the training data for the SD group was three times more than that of the non-SD group, a potential for training bias existed in this trial. The sensitivity and specificity predictions for most models were not biased toward the non-SD cluster (*p* = 0.08) and displayed no excessive discrepancy^30,33^.

The SD prediction performance was tested using the separately created test data, and the obtained results indicated the highest accuracy of the SVM model, with a sensitivity of 81.34% (CI 65.37–84.82), specificity of 87.97% (CI 68.6–99.4), PPV of 90.62% (CI 82.64–95.63), PNV of 69.77% (CI 52.99–83.85), and accuracy of 85.45% (CI 75.64–92.70), as illustrated in Fig. 4. In contrast, the DT model revealed the lowest performance with 80.69% sensitivity (CI 67.4–88.2), 74.92% specificity (CI 52.7–90.2), 86.72% PPV (CI 79.29–92.43), 54.58% PNV (CI 38.57–68.05), and 76.0% accuracy (CI 65.37–84.82). Notably, SVM, RF, DT, and LR delivered the highest accuracies. In the ROC analysis, SVM exhibited the highest AUC (0.88, CI 0.78–0.94), followed by LR (0.85, CI 0.75–0.92), RF (0.83, CI 0.73–0.90), and DT (0.81, CI 0.71–0.89). Overall, the variations between the AUCs (*p* = 0.41) were insignificant.

The heatmap effects of each feature on SD prediction for each model are illustrated in Fig. 5. Across all models, waist circumference had the greatest influence on SD prediction (RF = 0.15, SVM = 0.13, DT = 0.14, and LR = 0.13) and PSQI snoring loudness (RF = 0.04, SVM = 0.09, DT = 0.05, and LR = 0.13). Accordingly, the machine learning models developed for SD prediction were integrated into an application that yielded a probability for SD upon inputting these seven features.

Based on this outcome, we deduced that the training data accurately represented the SD and could be used in machine learning algorithms. The older SVM model outperformed the newer DT and RF models in this study. In general, the SVM performs appropriately with small datasets, which justifies its frequent and wide applications. However, SVM suffers from the limitation that it loses precision after a certain number of boundary overlaps^34,35^. Consequently, the number of knowledge points increases because the accuracy decreases when the boundary between the information for the prediction is uncertain. The extent of data was not sufficiently large to obtain reasonable performance because of the prominent deviation in the number of participants from the SD and non-SD groups^31^. Because the training dataset was small, a larger volume of data should be obtained in the future to ensure distinct characteristics between the SD and non-SD groups. Among the seven main features, waist circumference and PSQI snoring volume were the two characteristics that significantly affected SD prediction, with snoring being a vital sign of sleep apnea and one of the most noticeable signs of the disease.

As expected, snoring volume was directly correlated with the severity of SD (AHI). Notably, a large waist circumference is a significant risk factor and predictor of SD, as well as a significant contributor to the severity of the condition. In a previous study, this correlation was observed among Indians. Snoring volume and waist circumference have been highlighted as crucial factors in previous investigations of SD prediction models^36,37^. Based on relevant data from individuals suspected of SD from Asian countries, as well as the most recently developed algorithms, this study constructed SD prediction models and compared their performances to examine the most suitable machine learning model for predicting SD. Thus, machine learning models offer promising potential for predicting SD, as demonstrated by the high accuracy of the four machine learning models^38,39^. In particular, the SVM is the most effective model for predicting SD based on small datasets. However, this study has certain limitations, owing to the limited sample size. Owing to the magnitude of the biased information in the relationship between the SD and non-SD teams, a reasonable degree of uncertainty perturbed the model performance because the entire dataset was clearly insufficient for training and validating the machine learning models^40^.

In addition, overfitting was a limiting factor in validation during model training and substantiation. Moreover, there is a possibility of bias when using random-check information in the validation process^41^. Notably, the validation results differed completely when alternative randomly selected data were used for the testing. Nonetheless, overfitting was unlikely because of the limited size of the dataset, and the model performance remained consistent throughout the training and testing stages. However, validation requires additional overfitting analysis.

Thus, additional training data must be acquired for machine learning, and in the future, overfitting analyses should be conducted using validation methods, such as cross-validation and external validation. Further research on more extensive datasets and additional analyses will potentially improve the performance of the DT, RF, and SVM. Notably, machine learning algorithms such as LR, SVM, RF, and DT are considerably promising for the American state prediction of victimization data from India. Thus, machine learning is critical in the context of SD prediction. According to previous studies, the analytical technique is notable from the findings of construction workers. Instead of using LR or SVM to predict outcomes from American states, several machine learning methods can be applied, and their results can be comparatively analyzed to determine the most appropriate strategy for state prediction. The current SD prediction model achieved an AUC of 0.87 in comparison to a CAUC of 0.78 obtained by the LR-based SD prediction model of the Spanish cluster.

The SD prediction model constructed based on the responses from worker groups delivered an accuracy of 87.72% when compared to the current model, which displayed an accuracy of 83.33% and used a constant machine learning model similar to the SVM model used in this study. The South Indian state prediction model exhibited a sensitivity and specificity of 80.33% and 86.96%, respectively, using the same SVM model with marginal deviations and stable performance. In contrast, the American state prediction model proposed by the construction labor cluster exhibited an extremely large deviation between sensitivity and specificity of 42.86% and 94%, respectively, with an extremely low sensitivity. This indicated that the non-SD group received preferential treatment in terms of instruction or training and, as expected, learning did not occur. This outcome could be a result of the magnitude of the knowledge composition relationship between the SD and non-SD groups, along with unoptimized training parameters or methods for feature selection. Thus, a superior SD prediction model was preferred.

Compared with previous studies, the knowledge base for this research was smaller, which could be perceived as a drawback. Consequently, further studies are required to obtain more comprehensive data. In the future development of digital healthcare, machine learning approaches will be comparable to mobile applications for tailored observance of American states. More importantly, the daily progression of SD risk and AHI risk can be tracked using physiological data, such as atomic number 8 saturation, snoring sound, respiratory pattern, and pulse recorded during sleep using wearable devices or mobile phones. Furthermore, data related to cardiovascular disease and anthropometric parameters can be combined and analyzed using machine learning methods.

In summary, the key findings from this study demonstrate the efficacy of machine learning techniques, particularly the support vector machine (SVM) algorithm, in accurately predicting sleep deprivation among construction workers based on seven identified predictive factors. The SVM model achieved superior performance with 85.45% accuracy, 81.34% sensitivity, and 87.97% specificity, outperforming other models like random forest, decision tree, and logistic regression. Moreover, waist circumference and snoring loudness emerged as the most influential factors contributing to sleep deprivation prediction across all models. A subsequent study would aim to examine indicators such as feature importance and Shapley Additive exPlanations (SHAP) to assess their significance^42^.

## Conclusions

Sleep deprivation poses a significant challenge across various sectors, particularly in the construction industry. This issue not only impacts the health and safety of workers but also their overall job efficiency. Numerous studies have explored how lack of sleep affects safety and productivity, particularly through cognitive deficits. However, there is a notable scarcity of research on the identification and mitigation of workplace hazards. To address this research gap, the current study focuses on the application of machine learning algorithms to predict hazardous conditions. This particularly highlights the effectiveness of specific techniques, such as support vector machines and random forests, in predicting sleep deprivation among construction workers. Based on the data collected from 240 construction workers, 92 variables related to sleep deprivation were identified. Seven key indices were chosen for detailed analysis. This study developed and validated four types of machine learning models for predicting SD: SVM, RF, logistic regression, and DT. Using data from South Indian construction workers with suspected SD, all four models exhibited strong SD prediction performance, wherein the SVM yielded the best SD prediction result. Therefore, machine learning techniques are essential for developing a viable digital sleep health system to predict sleep deprivation and sleep disorders in the future.

The findings of this study have significant real-world implications and contribute to the academic discourse on construction safety and worker well-being. By developing and validating machine learning models for predicting sleep deprivation among construction workers, this research paves the way for practical applications that can proactively identify at-risk individuals and facilitate targeted interventions. Project managers and safety professionals can leverage these predictive models to implement tailored strategies, such as adjusting work schedules, providing sleep hygiene education, or offering counseling services, to mitigate the adverse effects of sleep deprivation. Moreover, the academic contribution of this study lies in its demonstration of the efficacy of machine learning techniques in addressing a critical issue within the construction industry, thereby expanding the knowledge base and fostering further research in this domain.

One of the limitations of this study was the relatively small sample size. To this end, future follow-up studies should be conducted on machine learning and artificial intelligence (AI) approaches for predicting SD in construction workers using extensive datasets to improve the performance of relevant machine learning methods. Specifically, future studies should investigate fitting deep learning models, such as convolutional neural networks, recurrent neural networks, long short-term memory, and deep neural networks, for structured data to predict worker sleep deprivation. The application of machine learning algorithms to predict sleep deprivation can significantly improve the health and safety of construction workers by identifying those at risk. The early detection of sleep deprivation can lead to interventions that prevent accidents and health issues. Additionally, mitigating the risks associated with sleep deprivation can enhance overall job efficiency and reduce the likelihood of accidents, which in turn can decrease costs related to workplace injuries and inefficiencies and improve overall construction project success.

## Data Availability

The corresponding author will provide data on request to support the findings of this study.
